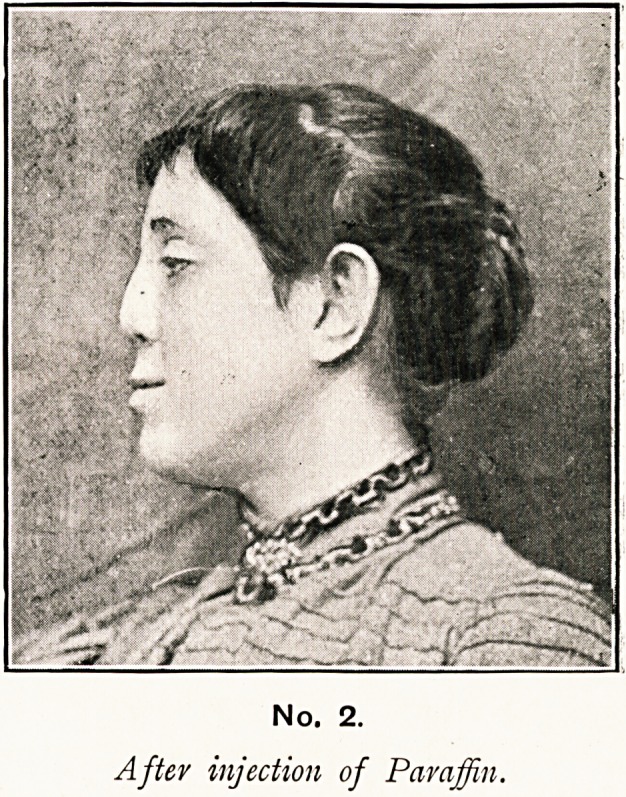# The Treatment of Certain Deformities, More Especially of the Nose, by the Subcutaneous Injection of Paraffin

**Published:** 1902-09

**Authors:** J. Paul Bush

**Affiliations:** Surgeon to the Bristol Royal Infirmary


### THE TREATMENT OF CERTAIN DEFORMITIES, MORE ESPECIALLY OF THE NOSE, BY THE SUBCUTANEOUS INJECTION OF PARAFFIN.

BY

J. PAUL BUSH, C.M.G.,

*Surgeon to the Bristol Royal Infirmary.*

---

THE subcutaneous injection of paraffin was brought to our notice by Gersuny, of Vienna, who, a year ago, published a series of 30 cases with no accident. It is, however, quite an open question whether or not this mode of correcting defor-

**No. 1.**

*Before Treatment.*

**No. 2.**

*After injection of Paraffin.*

mities will in the future be largely used, as we have yet to learn what percentage of failures takes place.

The "saddle-back" nose of inherited syphilis, where the lateral arches and the septum of the nose have ulcerated and left little else than the columnæ to support the tip of the nose, forms the larger class of deformities where this line of treatment may be considered. It may also be tried for certain forms of incontinence of urine, for incontinence of fæces, after excision of the rectum, or after extensive operation for anal fistula: I have myself used it for the latter condition with some little success. It has also been suggested to use it for defects after operation for cleft palate, for prolapse of the uterus in suitable cases, when a kind of subcutaneous pessary of paraffin is deposited, also after excision of the jaw to raise a depressed cheek, and many other conditions.

The skin of the nose is cleaned in a surgical manner, and a mixed paraffin (that is, a mixture of hard and soft paraffin in such proportions as ensures a melting point of 105° or 106° F.) having been carefully sterilised shortly before being used, is drawn into a large hypodermic syringe, and one to two drachms injected under the soft parts. Great care must be taken to prevent the injection travelling along the loose planes of connective tissue, and so into the eyelids: this is best done by the fingers of an assistant keeping up constant pressure till the paraffin becomes partially set. The paraffin must not be used too hot, as there is danger of causing sloughing or thrombosis in the neighbouring veins, and so pulmonary embolism. It usually sets up a mild reaction in the parts round the puncture for a day or two; granulations very slowly grow into the paraffin; at the end of a week it is still doughy, but getting firmer, and finally the mass becomes encapsuled and quite hard in the course of two months.

The paraffin does not appear to be absorbed for years, at any rate; for Gersuny found the mass undiminished in size two years after operation.

Out of three cases, I am only able to show two photographs of a girl of eighteen, who has been under my care for nine years for a gradual falling in of the bridge of the nose, due to destruction of the bony and cartilaginous supports by ulceration, which the mother states was due to a wound caused by falling on a knitting-needle which the child had in her mouth, and which perforated the nasal cavity.

No. 1 photograph, taken before operation, shows the marked "saddle-back" condition that existed; and No. 2 photograph, taken two months after operation, shows the marked improvement to the bridge of the nose.

Three months after operation there was some slight suppuration, or rather exudation of paraffin to a small extent; but this has ceased, and has not altered the shape of the nose as shown in photograph No. 2.

The second case was similar to the one described above, but with no suppuration; in the third patient the paraffin was injected in two places on each side of the anus in a case where there was some incontinence of fæces following an extensive operation for numerous fistulæ round the anus, which involved the removal of a considerable portion of the soft parts. The injection in this case caused no trouble, and has apparently been quite successful in its action.

## Figures and Tables

**No. 1. f1:**
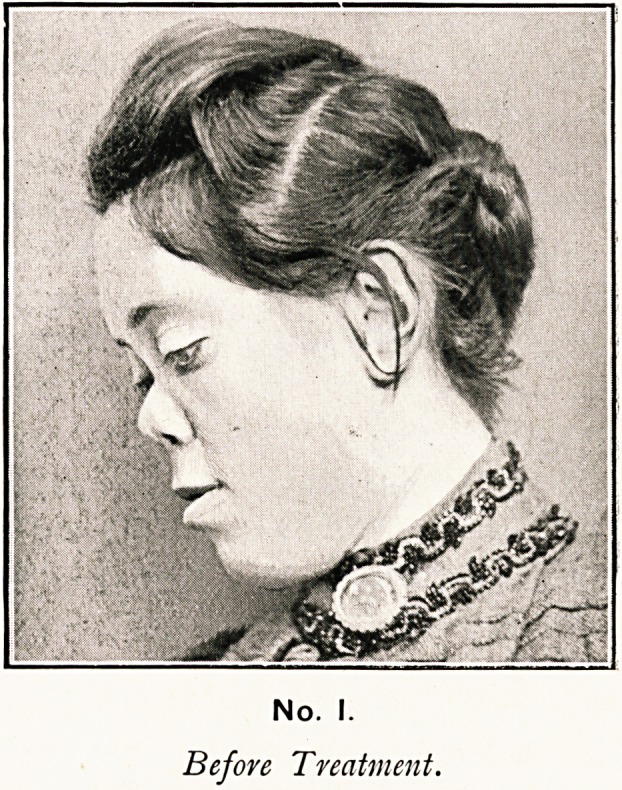


**No. 2. f2:**